# Tissue-specific transcriptome sequencing analysis expands the non-human primate reference transcriptome resource (NHPRTR)

**DOI:** 10.1093/nar/gku1110

**Published:** 2014-11-11

**Authors:** Xinxia Peng, Jean Thierry-Mieg, Danielle Thierry-Mieg, Andrew Nishida, Lenore Pipes, Marjan Bozinoski, Matthew J. Thomas, Sara Kelly, Jeffrey M. Weiss, Muthuswamy Raveendran, Donna Muzny, Richard A. Gibbs, Jeffrey Rogers, Gary P. Schroth, Michael G. Katze, Christopher E. Mason

**Affiliations:** 1 Department of Microbiology, University of Washington, Seattle, WA 98109, USA; 2 Washington National Primate Research Center, Seattle, WA 98109, USA; 3 National Center for Biotechnology Information, National Institutes of Health, Bethesda, MD 20894, USA; 4 Department of Physiology and Biophysics, Weill Cornell Medical College, New York, NY 10065, USA; 5 Institute for Computational Biology (ICB), Weill Cornell Medical College, New York, NY 10065, USA; 6 Human Genome Sequencing Center, Baylor College of Medicine, Houston, TX 77030, USA; 7 Illumina, Inc., San Diego, CA 92122, USA; 8 Feil Family Brain and Mind Research Institute (BMRI), Weill Cornell Medical College, New York, NY 10065, USA

## Abstract

The non-human primate reference transcriptome resource (NHPRTR, available online at http://nhprtr.org/) aims to generate comprehensive RNA-seq data from a wide variety of non-human primates (NHPs), from lemurs to hominids. In the 2012 Phase I of the NHPRTR project, 19 billion fragments or 3.8 terabases of transcriptome sequences were collected from pools of ∼20 tissues in 15 species and subspecies. Here we describe a major expansion of NHPRTR by adding 10.1 billion fragments of tissue-specific RNA-seq data. For this effort, we selected 11 of the original 15 NHP species and subspecies and constructed total RNA libraries for the same ∼15 tissues in each. The sequence quality is such that 88% of the reads align to human reference sequences, allowing us to compute the full list of expression abundance across all tissues for each species, using the reads mapped to human genes. This update also includes improved transcript annotations derived from RNA-seq data for rhesus and cynomolgus macaques, two of the most commonly used NHP models and additional RNA-seq data compiled from related projects. Together, these comprehensive reference transcriptomes from multiple primates serve as a valuable community resource for genome annotation, gene dynamics and comparative functional analysis.

## INTRODUCTION

Non-human primates (NHPs) are critical biomedical models for many aspects of human health and disease, but with much less available genomic resources compared to other model organisms. To develop a reference gene expression resource, the Non-Human Primate Reference Transcriptome Resource (NHPRTR, http://nhprtr.org) was initiated to deeply sequence RNA from multiple primates using next-generation sequencing (RNA-seq). In 2012, NHPRTR successfully produced and released reference transcriptome data for 15 NHP species/subspecies, which were collected for each species using pools of RNA from among 20 varieties of tissues (1).

As shown by our own analysis (2), thousands of un-annotated transcripts could be uncovered from the primate RNA-seq data released by NHPRTR Phase 1 data. However, the tissue-specific expression pattern of genes was not available, since those RNAs were pooled from different tissues before RNA-seq library preparation. Recently, several studies have shown that comparative analysis of tissue-specific RNA-seq data across different species is extremely valuable in studying the evolution of gene expression (3), splicing patterns of coding genes (4) and long noncoding RNAs (5), which likely contribute to the phenotypic differences between species. In addition, if one transcript is expressed only in one tissue, it will be diluted after pooling RNAs from different sources, and therefore might not be sequenced with a depth sufficient for transcript re-construction. RNA-seq analysis of each tissue individually is highly desirable, in terms of both comparative analysis and genome annotation.

Here we describe a new development of NHPRTR and make public the first large collection of tissue-specific primate transcriptomic data. For 11 of the original 15 NHP species/subspecies we prepared 9 to 15 tissue-specific RNA-seq libraries, depending on the availabilities of RNAs. We also added a small number of tissue-specific RNA-seq data from three more NHP species. The complete tissue-specific RNA-seq dataset consists of 157 libraries across 14 species/subspecies and over 10 billion read pairs totaling 2.44 terabases of Illumina sequence. We show the high quality and consistency of the whole dataset by aligning all RNA-seq reads to the same human reference sequences, followed by a thorough characterization including both expression pattern and genetic analysis. We also describe additional updates such as NHP tissue expression abundance, improved macaque annotations and additional RNA-seq resources. Together, with the expansion of these tissue-specific primate RNA-seq data, NHPRTR aims at providing a valuable resource to the community with comprehensive reference transcriptomes for each species.

## OVERVIEW OF TISSUE-SPECIFIC PRIMATE RNA-SEQ DATA

**Figure 1.**
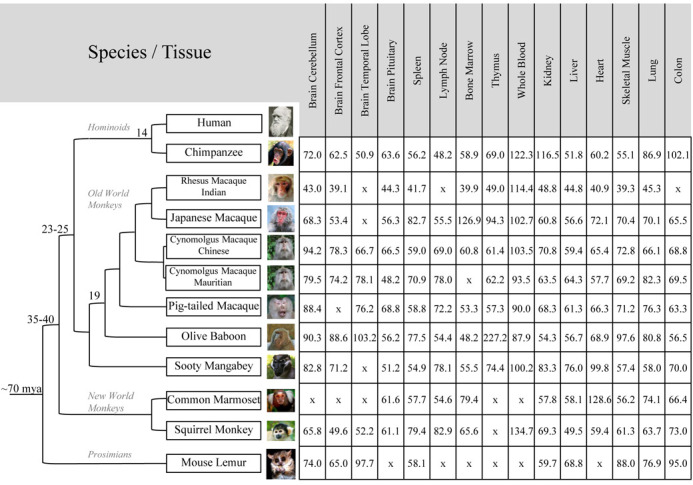
Summary of tissue-specific raw RNA-seq data (in millions of read pairs) for 11 NHP species and subspecies. All RNA-seq libraries were prepared with total RNAs with the strand-specific protocol and ribosomal RNA depletion (100 + 100 nt paired-end reads). Whole blood libraries were added a globin depletion step (50 + 50 nt paired-end reads). Additional tissue-specific RNA-seq data available from this NHPRTR update but not included in this table are: (i) testis (35.4 million read pairs) and ovary (33.1) from rhesus macaque Indian-origin; (ii) left (50.9) and right (69.5) brain hemispheres from marmoset; (iii) whole blood from ring tailed lemur (102.4), owl monkey (94.0) and rhesus macaque Chinese-origin (107.3); (iv) liver samples (50-nt single read reads) with polyA selection from Rhesus macaque Chinese-origin (26.2), cynomolgus macaque Mauritian-origin (20) and cynomolgus macaque Chinese-origin (16.9).

From the original collection of 21 tissues from 15 NHP species/subspecies, we selected 11 species/subspecies and 9 to 15 tissues from each species for extensive RNA-seq analysis (Figure 1). Besides the availability of sufficient amounts of good quality RNAs, the species and tissues were chosen to cover large evolutionary distances and to be most relevant to biomedical researchers. The tissues selected reflect the importance of particular primate models in the study of human diseases like AIDS pathogenesis and vaccine development, respiratory diseases, metabolic disorders and neurobiology. Specifically, we sampled five hematopoietic or immune system tissues (bone marrow, spleen, lymph node, thymus and whole blood), five central nervous system locations (cerebellum, frontal cortex, temporal lobe, global cortex and pituitary) and six main organs (kidney, liver, heart, skeletal muscle, lung and colon). We also sequenced ovaries and testes from Indian-origin rhesus macaque (RMI) and whole blood from ring tailed lemur, owl monkey and Chinese-origin rhesus macaque (RMC). Finally, as an internal control, liver samples from cynomolgus (Mauritian and Chinese, CMM and CMC) and Chinese-origin rhesus macaque were sequenced using a polyA selected protocol.

As shown in Figure 1, the full NHP tissue-specific dataset consists of 157 libraries from 14 species/subspecies; all but three used the strand-specific UDG (uracil-DNA glycosylase) protocol on total RNAs with ribosomal depletion; whole blood samples were additionally globin-depleted (prepared using the TruSeq Stranded Total RNA with Ribo-Zero Globin kit from Illumina). The entire NHP tissue-specific dataset includes 10.88 billion paired-end raw reads; all pairs are 100 + 100 nt, except for the whole blood libraries, which are 50 + 50 nt. On average, there are 66.8 million paired-end reads per tissue and species for non-whole blood samples (ranging from 42 million on average in RMI to 95 million in chimpanzee) and around 100 million paired-end reads for whole blood samples.

We took several measures to ensure the comparability of the data across NHP species and tissues. First, all RNAs were prepared by a single group using the same experimental protocol. Second, all sequencing libraries were prepared using the same experimental protocol (except for three additional liver controls) and sequencing was carried on the same Illumina HiSeq 2000 platform. Third, with the exception of Indian-origin rhesus macaque, all sequencing libraries were generated by a single group and sequenced in the same sequencing center. Indian-origin rhesus macaque samples were sequenced separately in order to facilitate the comparisons with additional RNA-seq data from individual macaques involved in on-going SIV/HIV related studies. In particular, as described below, one of the studies will perform RNA-seq analysis of sorted immune cell subsets from NHP species in the context of HIV/SIV infection.

The choice of total RNA-seq protocol was motivated by two major considerations. First, the RNA Integrity (RIN) values of RNAs from some NHP tissues were variable, and we and others have shown that RNA-seq analysis of total RNAs can accommodate poor-quality RNAs more efficiently (6,7), while oligo-dT selection used by standard mRNA-seq will only isolate the 3′-ends of degraded transcripts. Second, RNA-seq analysis of total RNAs also offers a broader coverage of transcriptomes compared to standard analysis of mRNAs, since total RNA-seq allows the detection of many non-polyadenylated transcripts, which include both coding genes like histones (8) and many long non-coding RNAs (2,7,9). By combining this collection of total RNA-seq data with previously collected mRNA-seq data, we expect an overall better coverage for transcript reconstruction.

## ASSESSMENT OF DATA QUALITY, SPECIES CONSISTENCY AND TISSUE-SPECIFICITY

**Figure 2.**
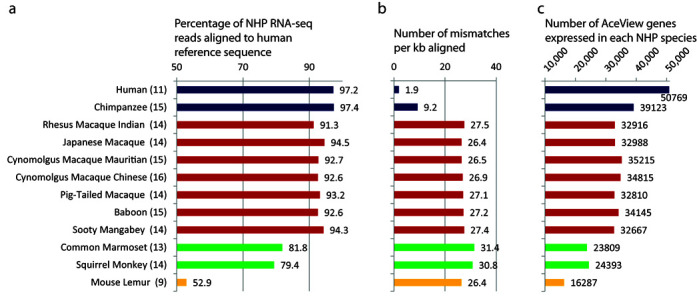
Summary of alignment of non-human primate (NHP) tissue-specific RNA-seq reads to human reference sequences. (**a**) Percentage of all reads from each NHP species which were aligned to human reference sequences using the Magic pipeline. The colors of bars indicate: Hominoids (blue), Old World monkeys (red), New World monkeys (green) and Prosimians (yellow); the number of tissues sequenced in each species is indicated in parenthesis. (**b**) Average number of mismatches per kilobase found in the alignments of each species to the human reference sequences. (**c**) Number of AceView genes with significant expression detected in at least one tissue of each primate species.

We evaluated the overall data quality using the Magic pipeline developed at NCBI (ftp://ftp.ncbi.nlm.nih.gov/repository/acedb/Software/Magic, Supplementary Material and (10,11)). Briefly, we mapped all NHP tissue-specific RNA-seq data to a collection of human reference sequences comprised of human genome (GRCh37) transcript sequences from RefSeq (http://www.ncbi.nlm.nih.gov/refseq and (12)) and AceView (http://www.aceview.org and (13)) (Supplementary Figure S1). This paradigm provided a common reference frame in which the expression level of the recognized counterparts of the human genes can be measured and compared across tissues in each species or across species, though with the caveat that the expression measures are influenced both by the abundance of the gene in the species and by its sequence conservation relative to human. However, as shown in Figure 2a and b, this did not appear to be a serious issue with apes and Old World monkeys, where at least 91% of all reads align with <28 mismatches per kb aligned. In New World monkeys, where ∼80% of the reads align with 31 mismatches per kb, we approach the recognition threshold of the aligner. There is no doubt that in lemurs, only the most conserved genes can be quantified with our procedure, since only 53% of the reads can be mapped to human with an apparent drop to 26 mismatches per kb, lower than in New World monkeys, indicating saturation. However, over 16 000 genes expressed in the lemur are still reliably identified and counterparts of over 32 000 human genes can be measured in New World monkeys. The relative expression of a given gene across the different tissues of a given species has no reason to be biased.

Overall, 19.3 (88.1%) of the 21.9 billion NHP RNA-seq reads were aligned to human reference sequences, indicating the good conservation of the genes across primates and the overall good sequence quality. Only 3.18% of all reads were mapped to ribosomal RNAs, showing the rRNA depletion protocol used was very efficient. As expected for RNA-seq analysis of total RNAs, many reads were mapped in part or as whole to introns, and overall only 49.15% map well completely inside the AceView genes and 41.65% inside the RefSeq genes. Altogether, there are 39 636 human AceView genes (and 136 901 distinct AceView transcripts) that are reliably expressed in the NHP tissues. Figure 2c shows the number of AceView genes detected in each species, and this inter-species differential has previously been used to characterize evolution of gene expression (14). The detailed quality control measures are described in the Supplementary Table S1.

**Figure 3.**
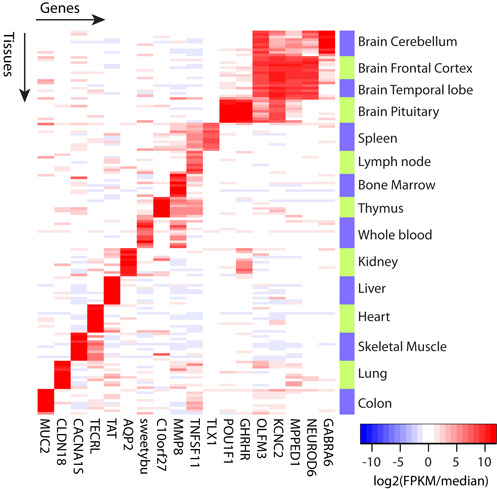
Examples of genes showing conserved tissue-specific patterns across NHPs. On the heatmap, columns are AceView human gene symbols and rows are individual tissue samples. Same tissues from different species were grouped together as indicated by the color bands and labels on the right side of the heatmap. The color on the heatmap indicates the relative gene expression abundance in individual tissue samples. The relative expression abundance was calculated as log 2 transformed normalized sFPKM subtracted by the median of log 2 transformed normalized sFPKMs across all tissue samples for the same gene, therefore a unit of 1 is equal to a 2-fold increase in abundance in a tissue compared to the median of all samples from the same tissue.

The species assignment of each library was verified by analyzing over 5 million positions which appear as homozygous variant relative to human in at least one species. The analysis was facilitated by the fact that in most cases, all tissues in a species were coming from a single individual. We observed that each library was overwhelmingly sharing its homozygous SNPs only with the other libraries of the same species (average 99.987% identical SNPs, range: 99.56 to 100.00%). Therefore, all species and subspecies are consistently annotated. The correct assignment of tissues was verified using the covariance analysis of the expression patterns provided by the Magic pipeline (Supplementary Methods). Examples of tissue-specific genes are shown in Figure 3.

To make this tissue-specific RNA-seq dataset more accessible, we created a master table providing the gene expression abundance of well annotated human genes in each of the 157 NHP tissue libraries, based on reads aligned to human reference sequences. For comparison, we also included 11 corresponding tissues from the Illumina 2010 Human BodyMap (http://www.ncbi.nlm.nih.gov/sra/?term=E-MTAB-513). It is worth noting that the calculated abundances could be confounded by sequence divergence, i.e. lower abundance could be attributed to low sequence similarity between human reference sequences and the particular NHP transcript sequences. Still this information may serve as a good starting point to browse genes which are highly conserved or which diverged from human, given the overall high sequence similarity between human and NHP species like rhesus macaques (15). Different versions of the full table of expression abundance of cognates of human genes based on mapped NHP RNA-seq reads are available on the updated NHPRTR website, which includes both raw read counts and normalized expression values (Magic expression index and significant FPKM) for both RefSeq and AceView annotated genes. The data can also be browsed gene by gene on the AceView website (http://www.aceview.org, Human Genes), where a color-coded matrix allows visual comparison of the level of expression of the gene across all primates and tissues (Supplementary Figure S2). From there, a pointer to the UCSC genome browser Magic hub gives access to the strand-specific coverage plots.

## ADDITIONAL UPDATES

Also available from this update is the improved annotation for Indian-origin rhesus and Mauritian-origin cynomolgus macaques, two of the most commonly used NHP models. Derived from the Phase I RNA-seq data, the improved annotation includes both thousands of novel isoforms for annotated genes and thousands of unannotated intergenic transcripts enriched with noncoding RNAs (2). We also identified thousands of transcript sequences that are partially or completely ‘missing’ from current macaque genome assemblies (2). Many newly identified transcripts were differentially expressed during Ebola virus infection of cynomolgus macaques (2) or simian immunodeficiency virus infection of rhesus macaques (2,16).

Further we have created a dedicated webpage pointing to additional related NHP transcriptome resources. In particular, we are interested in how NHPRTR data has been utilized by other resources and research. For example, Ensembl (http://www.ensembl.org and (17)) has used our baboon tissue-specific RNA-seq data to annotate the newly sequenced baboon genome. We provide a link to the RNA-seq read alignment files generated by Ensembl. Also, because the rhesus macaque is a widely used as a model to study HIV/AIDS, we provide a centralized pointer to macaque RNA-seq datasets released by the NHP Functional Genomics Core recently established by NIAID (http://nhp-fgc.org). This includes publically released data such as an mRNA-seq dataset of macaque rectal samples from multiple baseline and SIV-infected animals (16).

## FUTURE DIRECTIONS

We are actively working on RNA-seq analysis of NHP immune cell subsets. Although many investigators use NHP as models for AIDS pathogenesis, prevention and therapeutics, there is a lack of comprehensive datasets on the transcriptional profiles of various immune cell types from different NHP species—both at baseline and after HIV/SIV infection. This upcoming NHP immune cell RNA-seq dataset will cover sorted immune cell subsets (B cells, monocytes, NK cells, total CD4+ T cells, naïve CD4+ T cells, CD4+ central memory T cells, CD4+ effector memory T cells and CD8+ T cells) from rhesus macaque, African green monkey, sooty mangabey and human peripheral blood mononuclear cells, from both naïve, SIV/HIV acutely infected and chronically infected animals and individuals.

The main goal in generating these large datasets is to provide a rich resource for improving NHP gene models and for comparative analysis. To achieve this goal, we encourage the use of the data by the community, and we will continue assisting investigators in navigating, hosting and connecting additional NHP RNA-seq datasets. Together, these expanded NHP datasets serve as an NHP community resource and provide comprehensive reference transcriptomes for each NHP species.

## SUPPLEMENTARY DATA

Supplementary Data are available at NAR Online.
